# 

**Published:** 1889-03

**Authors:** 


THE OPEN COURT PUBLISHING CO., of Chicago, announces the appearance within the present month of an important contribution to Experimental Psychology, by the eminent French scientist, Alfred Binet. The work is entitled "The Psychic Life of Micro-Organisms," and is published with the sanction of the author, who has written a preface especially for the American edition.

The essays forming the work appeared originally in the *Revue Philosophique*, of Paris, and were afterwards published, in part, in *The Open Court.* The original cuts have been procured, and new plates and subsequent additions to the text have been incorporated in the work.

The monograph of M. Binet is a presentation of the most important results of recent investigations into the world of Proto-Organisms. M. Binet has added much to the psychology of the microscopic world by these researches; he has opposed many theories, confirmed others, and advanced many conclusions founded upon his personal investigation. The subject is a branch of comparative psychology little known and, as a rule, imperfectly understood. Psychologists and all who are interested in questions of biology, will accordingly look forward to the work of M. Binet as a welcome light on the problem of life. Price: cloth, 75 cents; paper, 50 cents. The Open Court Publishing Co., 169–175 La Salle street, Chicago, Illinois, P. O. Drawer F.

---

In Press—nearly ready—"American Resorts: with Notes Upon Their Climate." By Bushrod W. James, A. M., M. D., member of The American Association for the Advancement of Science; The American Public Health Association; The Pennsylvania Historical Society; The Franklin Institute, and The Academy of Natural Sciences, Philadelphia; The Society of Alaskan Natural History and Ethnology, Sitka, Alaska, etc. With a translation from the German, by Mr. S. Kauffman, of those chapters of "Die Klimate der Erde," written by Dr. A. Woeikof, of St. Petersburg, Russia, that relate to North and South America and the islands and oceans contiguous thereto. Intended for invalids and seekers after health and longevity, as well as for those who desire to preserve good health in a suitable climate. The American Biographical Publishing Co., Philadelphia, Pa., 1888.

---

Medical Books and Journals.—The remarkable catalogue of medical books and journals issued by Dr. A. E. Foot, of 1225 Belmont avenue, Philadelphia, has the same relation to the ordinary commercial catalogue as the Index Catalogue of the Library of the Surgeon General's office does to the ordinary catalogues. It is now being issued, being complete as to hygiene, yellow and other fevers; obstetrics, surgery, anatomy, practice, urinary, venereal, brain and nerve, etc. When fully completed, it will include over 14,000 titles. This remarkable catalogue is sent free to every physician requesting it on headed paper, or by including his professional card. It is indispensable to any practitioner who thinks or writes. This is, undoubtedly, the largest stock in the world. He issues similarly complete catalogues on chemistry, physics, and geology, and on all other branches of science. A natural taste for minerology has led Prof. Foot to accumulate the largest stock of minerals in the world—over 100 tons. These he sells at the ridiculously low price of $5 and $10 per 100 good specimens, carefully labeled; and 100 labeled crystals for $1. Price lists sent free.

---

THE WILLIAM F. JENKS PRIZE OF $250 has been awarded to Dr. John Strahan, of Belfast, Ireland, for the best essay on the "Diagnosis and Treatment of Extra-Uterine Pregnancy."

---

### BOOKS RECEIVED.

The Operations of Surgery: A Systematic Hand-book for Practitioners, Students and Hospital Surgeons. By W. H. A. Jacobson, F. R. C. S., Assistant Surgeon Guy's Hospital; Teacher Operative Surgery and Joint Teacher of Practical Surgery in the Medical School; Surgeon to the Royal Hospital for Children and Women. With 199 illustrations. Philadelphia: P. Blakiston, Son & Co., 1012 Walnut street. 1889.

Essentials of Physics and Chemistry, written especially for the use of Students in Medicine. By Condict W. Cutler, M. S., M. D. Third edition; enlarged and revised. Pp. 296. New York and London: G. P. Putnam's Sons. 1889. The Knickerbocker Press.

The Theory and Practice of Obstetrics, including Diseases of Pregnancy and Parturition, Obstetrical Operations, etc. By P. Cazeaux. Remodeled and rearranged with additions and revisions. By S. Tarnier. The eighth American edition, revised and edited by Robert J. Hess, M. D., with an appendix by Paul F. Mundé, M. D., with chromo-lithographs, lithographs and other full-page plates, and 175 wood engravings. Philadelphia: P. Blakiston, Son & Co., 1012 Walnut street. 1889.

Report of the Commissioner of Education for the Year 1886–7. Department of the Interior, Bureau of Education. By N. H. R. Dawson, Commissioner. 8vo, pp. 1,170. Washington: Government Printing Office. 1888.

Transactions of the American Gynecological Society. Vol. XIII. For the year 1888. 8vo, pp. 502. Philadelphia: Wm. J. Dornan, Printer. 1888.

Electricity in the Diseases of Women, with Special Reference to the Application of Strong Currents. By G. Betton Massey, M. D., Physician to the Nervous Department of Howard Hospital; Member of the American Neurological Association; of the Obstetrical Society, of Philadelphia, etc., etc. 12mo, pp. 210. Philadelphia and London: F. A. Davis, Publisher. 1889.

International Pocket Medical Formulary, with an Appendix containing Posological Table, etc. By C. Sumner Witherstine, M. S., M. D., Associate editor *Annual of the Universal Medical Sciences*, etc. Philadelphia and London: F. A Davis, Publisher. 1889.

### PAMPHLETS RECEIVED.

Some Clinical Experiences with Insomnia. By Edward N. Brush, M. D., Senior Assistant Physician, Pennsylvania Hospital for the Insane, Philadelphia; late Senior Assistant Physician, New York State Lunatic Asylum, Utica, N. Y.; formerly Visiting Physician, Hospital Sisters of Charity, Buffalo, N. Y. Reprint from *The Practitioner*, London.

Note on Rumbold's Method of Treatment of Catarrhal Inflammation of the Upper Air-Passages. By Ely McClellan, M. D., Surgeon United States Army. Reprint from the *Journal of the American Medical Association*, January 5, 1889.

Bureau of Education. Circular of Information No. 3. The History of Education in North Carolina. By Charles Lee Smith, Fellow in History and Politics, Johns Hopkins University. Washington: Government Printing Office. 1888.

Poisoning by Chrome Yellow used as a Cake Dye. The Subsequent History of the Cases, including a Case of Paralysis Agitans and of Chronic Endocarditis. By David Denison Stewart, M. D., Chief of the Medical Clinic of the Jefferson Medical College; Physician to St. Christopher's Hospital for Children. Reprint from the *Medical News*, January 26, 1889.

Lactucarium Syrup and Paste of H. Aubergier, Honorary Dean of the Faculty of Sciences; President of the Academy of Sciences, Belles-Lettres and Arts, of Clermont-Ferrand, etc., etc. E. Fougera & Co., New York. 1888.

The Immediate Application of Forceps to the After-Coming Head, in Cases of Version with Partial Dilatation of the Cervix. By H. C. Coe, M. D., M. R. C. S., New York. Reprint from the *Medical Record*, January 19, 1889.

Report of the Committee on Ophthalmology and Otology. By Seth S. Bishop, M. D., Chicago. Reprint from the Transactions of the Thirty-seventh Annual Meeting of the Illinois State Medical Society. 1888.

A Defence of Electrolysis in Urethral Strictures, with Documentary Evidence. By Robert Newman, M. D., New York, Surgeon to North-western Dispensary, N. Y.; Consulting Surgeon, Hackensack Hospital, etc., etc. Reprint from the *Medical Register*, January 5, 1889.

The Infertility of Women. The Nervous System in Sterility. Some of the Requirements of Treatment. By Henry F. Campbell, M. D., Augusta, Georgia. Reprint from Vol. XIII. Gynecological Transactions. 1888.

Food versus Bacilli in Consumption (opus 286). An Open Letter from Ephraim Cutter, M. D., LL.D., Corresponding Member Société Belge de Microscopie; Associate Member Philosophical Society of Great Britain, etc., etc., to his son, John Ashburton Cutter, M. D., B. Sc., with answer. New York: The Ariston. Published by the author. 1888. All rights reserved.

The Electrolytic Decomposition of Organic Tissues. By George H. Rohé, M. D., Professor of Dermatology and Hygiene, in the College of Physicians and Surgeons, Baltimore. Reprint from the *New York Medical Journal* for December 1, 1888.

Twelve Months of Abdominal and Vaginal Section. By Henry T. Byford, M. D. Presidential Address delivered at the annual meeting of the Gynecological Society of Chicago, October 19, 1888. Reprint from the *Chicago Medical Journal and Examiner*.

Prevention of Yellow Fever in Florida and the South. Read before the Baltimore Academy of Medicine. By W. C. Van Ribber, A. M., M. D., Baltimore. *Journal* Publishing Company print. 1889.

